# Ultrahigh performance supercapacitors utilizing core–shell nanoarchitectures from a metal–organic framework-derived nanoporous carbon and a conducting polymer†
†Electronic supplementary information (ESI) available. See DOI: 10.1039/c6sc01429a


**DOI:** 10.1039/c6sc01429a

**Published:** 2016-06-10

**Authors:** Rahul R. Salunkhe, Jing Tang, Naoya Kobayashi, Jeonghun Kim, Yusuke Ide, Satoshi Tominaka, Jung Ho Kim, Yusuke Yamauchi

**Affiliations:** a Mesoscale Materials Chemistry Laboratory , World Premier International (WPI) Research Center for Materials Nanoarchitectonics (MANA) , National Institute for Materials Science (NIMS) , 1-1 Namiki , Tsukuba , Ibaraki 305-0044 , Japan . Email: Yamauchi.Yusuke@nims.go.jp; b Faculty of Science and Engineering , Waseda University , 3-4-1 Okubo , Shinjuku , Tokyo 169-8555 , Japan; c TOC Capacitor , 3-20-32 Tenryucho , Okayashi , Nagano 394-0035 , Japan; d Institute for Superconducting & Electronic Materials , Australian Institute of Innovative Materials , University of Wollongong , Innovation Campus , Squires Way , North Wollongong , NSW 2500 , Australia . Email: jhk@uow.edu.au

## Abstract

Hitherto, many reports on composite materials for electrochemical applications are based on one-dimensional carbon nanotubes or two-dimensional graphene materials. However, these composite materials usually suffer from a stacking problem during electrochemical cycling. A smart nanoarchitectural design is needed for composite materials in order to overcome this problem. Recent research on electrochemical energy storage (EES) applications has focused on the development of three-dimensional (3-D) core–shell structures. The basis for high performance electrochemical energy storage is to control the efficient intercalation of ions in such a 3-D structure. Here, we demonstrate controlled synergy between the physicochemical properties of nanoporous carbon and conducting polyaniline polymer (carbon–PANI), which leads to some new interesting electrochemical properties. The time-dependent controlled optimization of the core–shell nanocomposites consisting of nanoporous carbon with a thin layer of PANI nanorod arrays gives useful control over supercapacitor performance. Furthermore, these carbon–PANI nanocomposites can electrochemically access ions with remarkable efficiency to achieve a capacitance value in the range of 300–1100 F g^–1^. When assembled in a two electrode cell configuration, the symmetric supercapacitor (SSC) based on carbon–PANI//carbon–PANI shows the highest specific energy of 21 W h kg^–1^ and the highest specific power of 12 kW kg^–1^. More interestingly, the SSC shows capacitance retention of 86% after 20 000 cycles, which is highly superior compared to previous research reports.

## Abstract 2

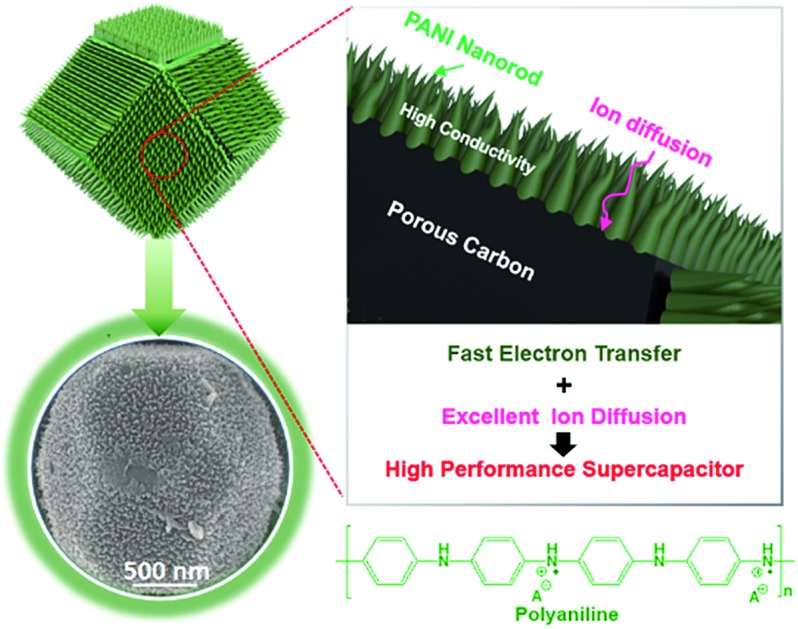
Nanoarchitectured nanoporous carbon/conducting polymer core–shell nanocomposites (carbon–PANI) are prepared from metal–organic framework-derived carbon and the controlled polymerization of polyaniline nanorods.

## Introduction

Since the last decade, many research efforts have been directed towards the development of three-dimensional (3-D) electrodes for electrochemical energy storage (EES) applications.^1^–^4^ This is because they offer a number of advantages, such as increased mass loading per unit area and improved electron and ion transportation through the porous network, as well as better mechanical stability over repeated cycle operations.^3^–^7^ The ideal 3-D structured electrode for EES applications is comprised of a core–shell architecture consisting of nanoporous carbon as the core with easy ion diffusion and a conductive redox active material as a shell with high electrical conductivity. For such a core–shell 3-D structure, it is possible to combine two different functionalities into one, where their functions can be operated independently but still perform synergistically.^8^ Such smart material design can bridge the gap between supercapacitors with high power densities and battery materials with high energy densities. The structural stability over repeated charge–discharge cycles is also an important factor for practical applications. Although several promising 3-D electrodes have been reported very recently, their lifespan is very limited.^1^

So far, many different types of nanoporous carbon materials have been employed for EES applications, owing to their unique properties such as high specific surface areas, good electrical conductivities, controlled pore structures, good thermal and mechanical stabilities, and relatively low costs.^9^ Among carbon allotropes, graphite, consisting of sp^2^-bonded carbon atoms, gives high conductivity because the electrons associated with the π-bonds become available to carry the charge.^10^ Surface functional groups and the degree of graphitization also play important roles in increasing the overall EES performance of the carbon.^11^ Additionally, a recent study has highlighted that, in order to achieve high energy density as well as high power density, the presence of both micropores and mesopores is very important.^12^ Micropores (generally less than 2 nm) are important for achieving high capacitance values and thereby high energy density, while mesopores (generally from 2 nm to 50 nm) can provide ions with easy access to the surface, and thereby high power density. Most micropores are inaccessible at high scan rates in aqueous electrolyte, which results in the slow diffusion of ions in micropores at high scan rates.^12^ Metal–organic framework (MOF) derived nanoporous carbons (including zeolitic imidazolate framework (ZIF) derived nanoporous carbons) have gained much attention due to their intriguing micro-meso porous architectures, their high surface areas (>1000 m^2^ g^–1^) and their moderate electrical conductivities.^13^–^17^ However, studies on electrical-double-layer capacitors (EDLCs) that take advantage of the performance of ZIF-derived nanoporous carbons are still limited.

On the other hand, conductive redox polymers, such as polyaniline (PANI), have proven to be promising electrode material for high capacitance applications, however they always suffer from limited stability.^18^ There are many reports available in the literature on the development of PANI as well as PANI-based nanocomposites using different synthetic routes for EES applications.^19^,^20^ A recent study has demonstrated the importance of obtaining the shortest diffusion paths for ordered PANI structures for better utilization of the high surface area as well as for improvement of the power density.^21^,^22^ Several composites of PANI with different carbon materials, such as one-dimensional (1-D) carbon nanotubes/nanofibers^23^–^29^ and two-dimensional (2-D) graphene,^30^–^38^ have been reported so far. The main disadvantage of such a 1-D or 2-D structure, however, is that stacking of layers can easily occur before or during electrochemical cycling, leading to blocking of ions by surface layers, resulting in poor cycling stability.

**Scheme 1:**
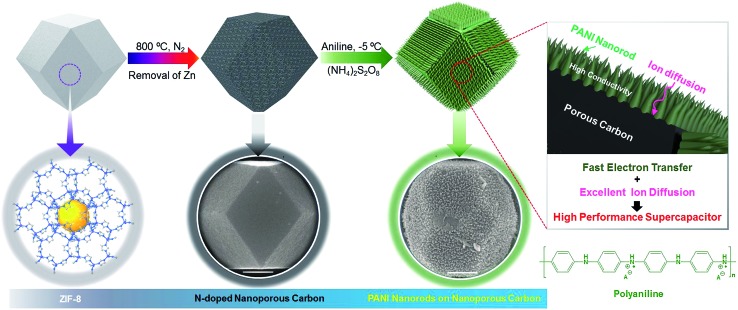
Schematic illustration of the synthetic process for the attainment of nanoporous carbon–PANI core–shell nanocomposite materials, starting from the rhombic dodecahedron ZIF-8. For the preparation of nanoporous carbon, ZIF-8 was carbonized and Zn was removed by washing with HF. The SEM images of the bare carbon and carbon–PANI nanocomposite are as shown. The scale bars are 500 nm in length. The PANI nanorods were grown on nanoporous carbon and the lengths of the nanorods were controlled by the polymerization time.

To further improve the EES performance, a well-designed composite material containing nanoporous carbon as the core and ordered polymer arrays as the shell is an ideal architecture. The advantage of this configuration is that it can improve the mechanical stability of the polymer without hampering the electronic conductivity of the carbon core, and it also provides the shortest diffusion path to the core (Scheme 1). Although a few reports are available on the development of core–shell composites of nanoporous carbon and PANI,^11^,^22^ sophisticated hybridization of nanoporous carbon with PANI nanorods has been never reported.

Herein, we present a facile controlled synthesis of core–shell nanocomposites (carbon–PANI) by chemical oxidative polymerization. Nanoporous carbon prepared from ZIF-8 was selected as the core for PANI nanorod growth (Scheme 1). By tuning the PANI shell thickness, we can control the synergy of these nanocomposites to achieve high capacitance performance. Finally, supercapacitor devices were successfully fabricated using three different symmetric configurations, carbon//carbon, PANI//PANI, and carbon–PANI//carbon–PANI. These three systems were compared to each other in terms of their specific energies and specific powers. The specific energy obtained in the present study is about ten times higher than that of commercially available activated carbon. This novel carbon–PANI electrode exhibits high capacitance retention as well as high specific capacitance.

## Experimental

### Synthesis of ZIF-8 derived carbon

For the synthesis of ZIF-8, 2-methylimidazole was dissolved in methanol in a beaker and stirred to prepare a clear transparent solution. In another beaker, Zn(CH_3_COO)_2_ was dissolved in polyvinylpyrrolidone (PVP) and this solution was stirred for 30 min to obtain a clear transparent solution. Then, these two solutions were mixed together and stirred for another 20 min. The resultant solution was aged for about 20 hours. After that, a white-coloured precipitate was observed at the bottom of the beaker. This precipitate was collected and dried for 12 h. In order to convert this ZIF-8 sample into nanoporous carbon, the sample was directly carbonised at 800 °C under a nitrogen atmosphere with a heating rate of 5 °C min^–1^ for 4 h. The carbon samples were washed with HF four times in order to remove Zn species and they were then used directly for further use.

### Polymerization of PANI and preparation of PANI-grown ZIF-8 derived carbon

For the PANI polymerization process, 30 ml of HClO_4_ (1 M) was mixed with ethanol (10 ml) in a beaker. This solution was stirred for about 10 min. After adding aniline monomer (0.05 M), the solution was stirred for about 30 min at –5 °C. Meanwhile, in another beaker, 0.03 M ammonium persulfate ((NH_4_)_2_S_2_O_8_) was added to 10 ml of 1 M HClO_4_ and the solution was kept at –5 °C, before being used as an oxidant in the reaction. The above two solutions were mixed together with stirring and the polymerization of PANI started with a slow reaction after 1 h at –5 °C. Thus obtained PANI was washed with ethanol and distilled water several times, and then dried and stored for further use.

To grow the PANI nanorods on nanoporous carbon, 9 mg of nanoporous carbon was added to a monomer-dissolved solution and the resulting solution was stirred, affording a uniform coating of monomer on the carbon surface. Then, the polymerization was carried out by adding the oxidant solution. The PANI chains result in one-directional growth of nanorods on the nanoporous carbon surface. The polymerization times were gradually varied from 1 h up to 5 h to control the thickness of the PANI shell. After polymerization, the obtained powder was washed with ethanol and distilled water several times and collected by centrifugation.

### Material characterization

The surface morphologies and structures of the obtained samples were characterized by scanning electron microscopy (SEM, HITACHI SU8000) and transmission electron microscopy (TEM, TECNAI 3010). Powder X-ray diffraction (XRD, Rigaku Rint 2500, Cu Kα, *λ* = 1.5406 Å) was used in order to determine the crystalline structures of the samples. The details of the different bonds present on the surfaces of these materials were characterized using FTIR (Nicolet 4700) and a micro-Raman (Horiba-Jobin Yvon T65000) analysis station. N_2_ adsorption–desorption isotherms were obtained using a BELSORP-max instrument.

### Electrochemical analysis

Electrochemical analysis was carried out using cyclic voltammetry (CV) and galvanostatic charge–discharge (CD) measurements. Furthermore, galvanostatic CD studies were carried out using a symmetric configuration. For this purpose, 1 M H_2_SO_4_ was used as an electrolyte. All of the electrochemical studies were carried out using an electrochemical workstation (CHI 660E, CH instruments USA). The carbon–PANI composite samples were mixed with poly(vinylidene difluoride) (PVDF, 20%) in *N*-methyl-2-pyrrolidone (NMP) as a solvent. The resulting slurry was homogenized by ultrasonication and coated onto a graphite substrate, which was then used as the working electrode (with the graphite substrate serving as the current collector). Each electrode contained 0.5 mg cm^–2^ of electroactive material. The SSC cells were fabricated from carbon–PANI composites with similar weight loadings. A two electrode cell containing positive and negative electrodes with a separation of 0.3 cm was used to test the electrochemical properties of the symmetric cell. For comparison purposes all of the electrochemical tests were carried out in the same potential window of 0.0 to 0.8 V. The CV and galvanostatic CD results were collected using an electrochemical workstation (CHI 660 E).

Specific capacitance values were calculated from the CV and galvanostatic CD results using the following equations:
1

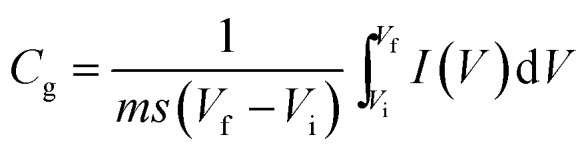



2

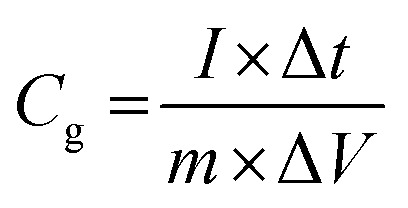


*I*: current (A), *m*: mass of the active electrode (g), *s*: scan rate (V s^–1^), Δ*V* = *V*_f_ – *V*_i_: potential window (V), Δ*t*: discharge time (s), and *C*_g_: gravimetric capacitance (F g^–1^).

The specific energy (SE) and specific power (SP) of the supercapacitors were calculated according to the following equations:
3

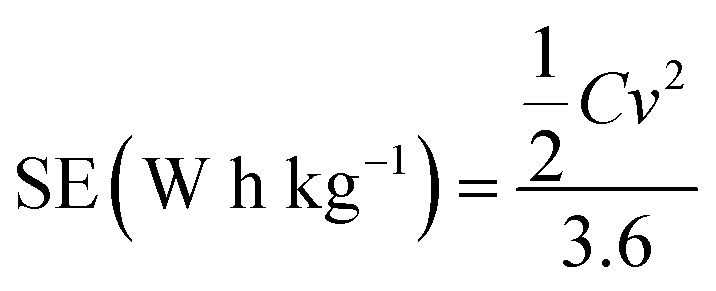



4

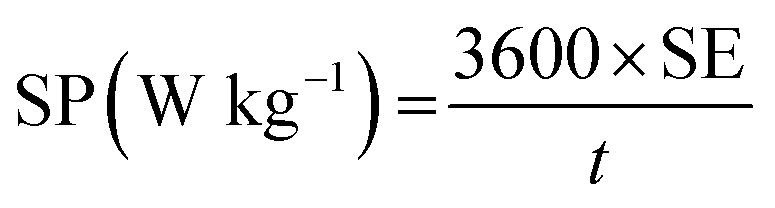


*C*: the total capacitance of the two electrode cell, *v*: the effective potential range during the discharging process, and *t*: the discharging time (s).

## Results and discussion

### Preparation and characterization of carbon–PANI composites

The synthesis of PANI was carried out through an oxidative polymerization, which is a typical method for PANI synthesis. The polymerization was performed by HClO_4_ and ammonium persulfate in the presence of aniline monomer. The prepared PANI was used and compared with nanoporous carbon–PANI composites. On the other hand, nanoporous carbon was prepared through the thermal treatment of ZIF-8 in nitrogen atmosphere. Studies comparing the porosity of our carbon sample with that of commercially available activated carbon were carried out (Fig. S1 in the ESI†). The Brunauer–Emmett–Teller (BET) surface areas are 2370 m^2^ g^–1^ and 1610 m^2^ g^–1^ for activated carbon and our carbon sample, respectively. The relative micropore ratio of the activated carbon sample is higher than that of our carbon sample (detailed analysis data is tabulated in Table S1†). For the preparation of nanoporous carbon–PANI composites, 9 mg of nanoporous carbon was added to a monomer-dissolved solution. The polymerization times were gradually varied from 1 h (S1) to 2 h (S2), 3 h (S3), 4 h (S4) and 5 h (S5) to control the thickness of the PANI shell. All details of material preparation are described in the Experimental section.

**Fig. 1:**
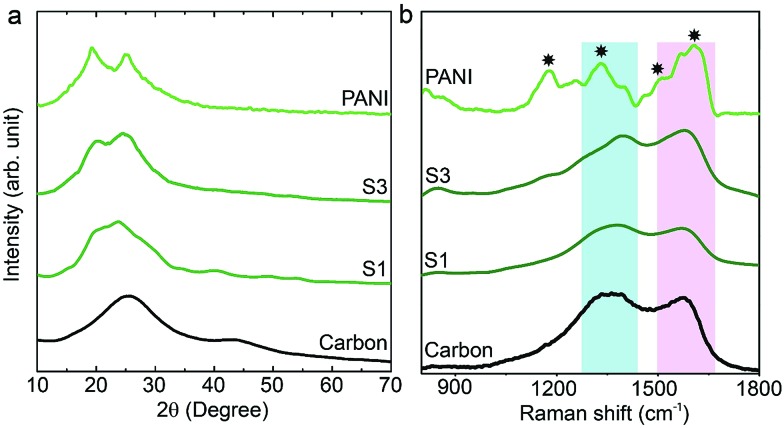
XRD and Raman characterization of the samples. (a) Wide angle XRD patterns of carbon, PANI and carbon–PANI nanocomposites (S1 and S3). (b) Raman spectra for carbon, PANI and carbon–PANI nanocomposites (samples S1 and S3).

Wide angle X-ray diffraction (XRD) measurements were carried out on the carbon, PANI and carbon–PANI composite samples. As seen from Fig. 1a, in the case of the carbon sample, the peaks at 25° and 44° are associated with the (002) and (101) interlayer peaks, characteristic of graphite-type carbon.^13^ The XRD peaks at 19° and 25° are the characteristic peaks of PANI.^37^,^39^ As the reaction time increases from sample S1 to sample S5, the peak intensity at 25° gradually increases. This peak at 25° is associated with the π–π interchain stacking distance between the phenyl rings. The increase in this peak intensity can indicate improved conductivity.^40^

Raman scattering can give more detailed information on the chemical bonds present at the surface (Fig. 1b). For the carbon sample, two peaks at 1591 cm^–1^ (G band) and 1351 cm^–1^ (D band) are observed, which correspond to graphitic carbon and disordered carbon, respectively.^41^ The corresponding peaks for both carbon–PANI composite samples are basically similar to those observed in the carbon sample, but the peak intensities and peak positions are slightly different as a result of the time-dependent coating of PANI. For the PANI sample, four major peaks can be observed (marked by asterisks in Fig. 1b). These are (1) C–H bending of the quinoid ring at 1176 cm^–1^, (2) C–N˙^+^ stretching vibration of cation radical species at 1332 cm^–1^, (3) C–C stretching of the quinoid ring at 1497 cm^–1^, and (4) C–C stretching of the benzene ring at 1615 cm^–1^, and they are characteristic of the presence of PANI.^42^ As the shell thickness increases (*e.g.* from S1 to S3 in Fig. 1b), the intensity in the range of 1500 to 1600 cm^–1^ also increases, due to overlap with the peaks derived from PANI. Furthermore, as the shell thickness increases, the peak at 1332 cm^–1^ corresponding to the C–N˙^+^ stretching vibration in PANI shifts to the higher wavenumber of 1360 cm^–1^. This is due to the strong interaction of the C–N˙^+^ species of PANI with a negative surface species of carbon (COO^–^).^42^ Also, this phenomenon can be more clearly observed with an increased coating time. Thus, peak shifting observed in the range of 1300 cm^–1^ to 1400 cm^–1^ is the result of the combined contribution of the carbon peaks and PANI peaks. Increasing the intensity (*I*) ratio *I*_C–N^˙+^_/*I*_C–H_ can promote protonic acid doping of the material.^43^ Thus, the XRD and Raman analysis give a clear indication of the successful formation of PANI with good properties.

**Fig. 2:**
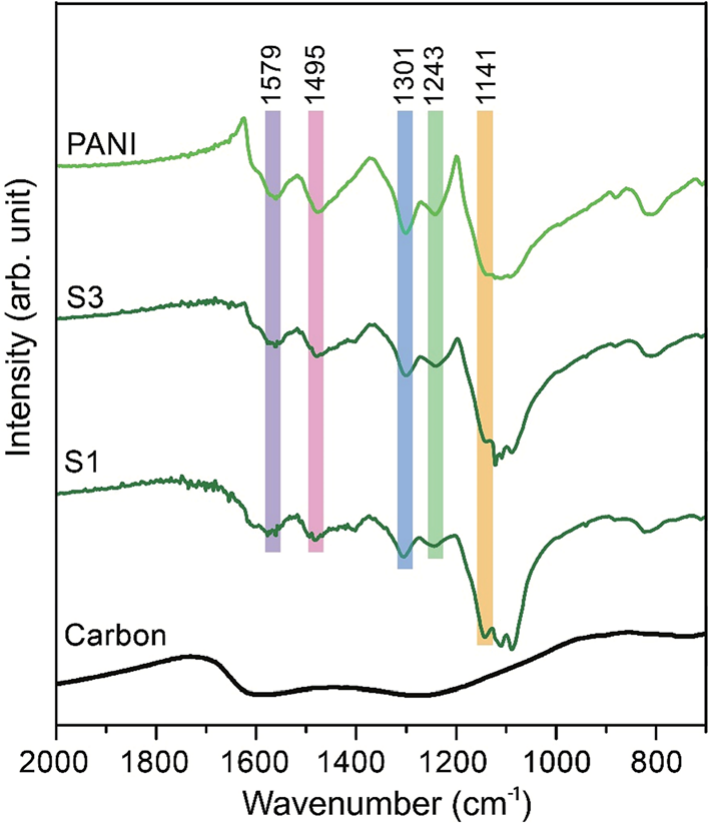
Fourier transform infrared (FTIR) studies for the samples. FTIR spectra of carbon, PANI and carbon–PANI composites (samples S1 and S3).

The Fourier transform infrared (FTIR) spectra of carbon, PANI and carbon–PANI nanocomposites are shown in Fig. 2. For the carbon sample, the C=N bond (at approximately 1600 cm^–1^) and N–H bonding (at approximately 1300 cm^–1^) show that forms of nitrogen bonding are present on the carbon surface.^44^ For PANI and carbon–PANI composites, the adsorption bands at 1495 cm^–1^ and 1579 cm^–1^ can be assigned to the C=C stretching vibration modes of the benzenoid and quinonoid rings of PANI, respectively.^45^ The adsorption band that appears at 1301 cm^–1^ is related to C–N stretching modes, whereas the adsorption band appearing at 1141 cm^–1^ is associated with the C–H in-plane bending mode of pernigraniline.^45^ Furthermore, a small adsorption band at 1243 cm^–1^ is associated with the protonated C–N^+^ group. Thus, it is proven that the PANI shell was successfully formed on the carbon surface.

**Fig. 3:**
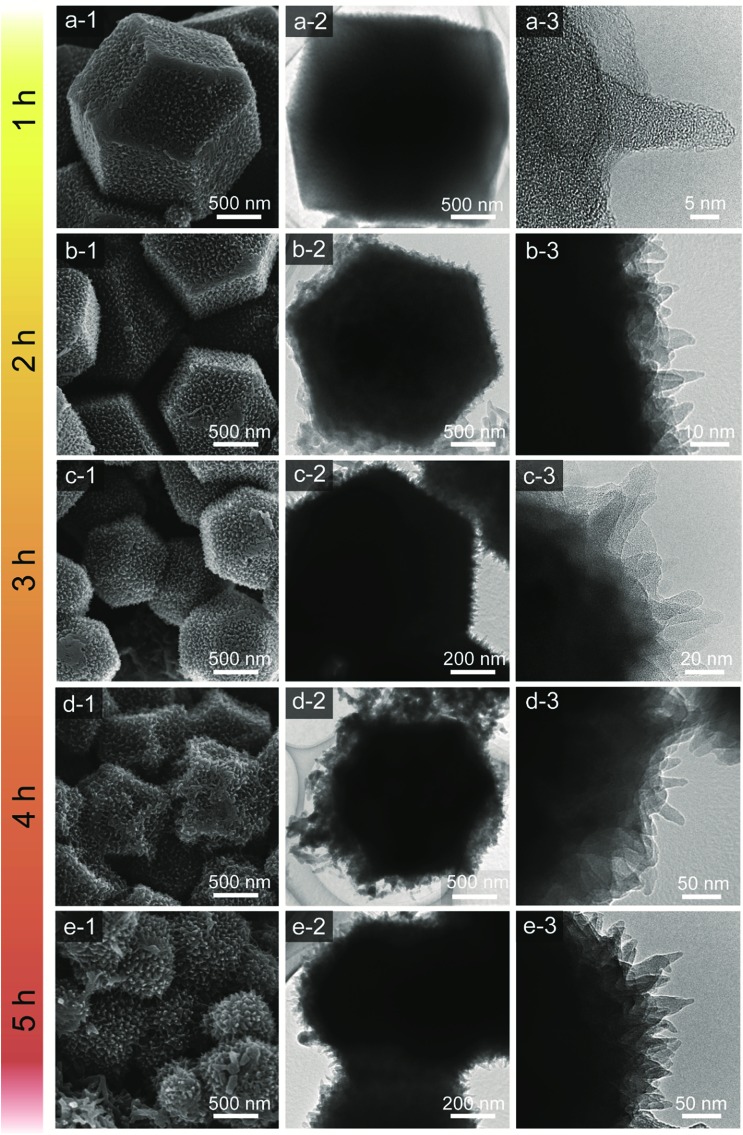
SEM (left column) and TEM images (middle and right columns) of carbon–PANI composites. (a1–a3) Carbon–PANI composites after a 1 h reaction time (sample S1); (b1–b3) after a 2 h reaction time (sample S2); (c1–c3) after a 3 h reaction time (sample S3); (d1–d3) after a 4 h reaction time (sample S4) and (e1–e3) after a 5 h reaction time (sample S5). Smaller sized particles were intentionally selected in order to clearly show the coating of the PANI nanorods on the particles.

Scanning electron microscopy (SEM) and transmission electron microscopy (TEM) were employed to study the morphologies of the carbon–PANI samples. For comparison, pure PANI was prepared without carbon supports. The complex nanofiber morphology can be confirmed by SEM and TEM images, as shown in Fig. S2.† Fig. 3 shows the time-dependent morphological changes in the PANI nanorod arrays on the carbon surface. By increasing the deposition time, the lengths and widths of PANI nanorods increase progressively. The low magnification SEM images of our carbon and carbon–PANI composites (S1–S5) are shown in Fig. S3.† At the early stage of PANI deposition, a very thin layer of oriented PANI gets coated on the carbon surface (Fig. 3a). This is because the nanoporous carbon surface provides abundant nucleation sites for the uniform growth of PANI nanorods. The TEM image shows that the length of these nanorods is about 10 nm, but the density of these nanorods is low. With increasing PANI deposition time (samples S2 and S3), uniformly aligned nanorods can be observed (Fig. 3b–c). The TEM image for sample S2 reveals that the width of the nanorods is almost 10–12 nm, and that the nanorods start to grow more densely. As shown in Fig. 3c, the average length of the nanorods also increased up to about 20–25 nm. Interestingly, the original polyhedral shape of the nanoporous carbon is totally retained, which can completely avoid the particle aggregation that has been commonly seen in previous research reports on carbon–PANI composites.^24^,^46^ With a further increase in the reaction time (4 h), the PANI nanorods become very dense due to agglomeration (Fig. 3d). The average length of the PANI nanorods is about 50 nm for sample S4. Although such a dense structure sets the limit for accurately measuring the nanorod lengths, a further increase in the reaction time leads to an increase in the length of the PANI nanorods (Fig. 3e). To confirm the presence of carbon and nitrogen in the above samples, TEM elemental analysis was carried out (Fig. S4†), and carbon and nitrogen were confirmed to be present in both the carbon and carbon–PANI samples.

### Electrochemical capacitance of carbon–PANI composites

**Fig. 4:**
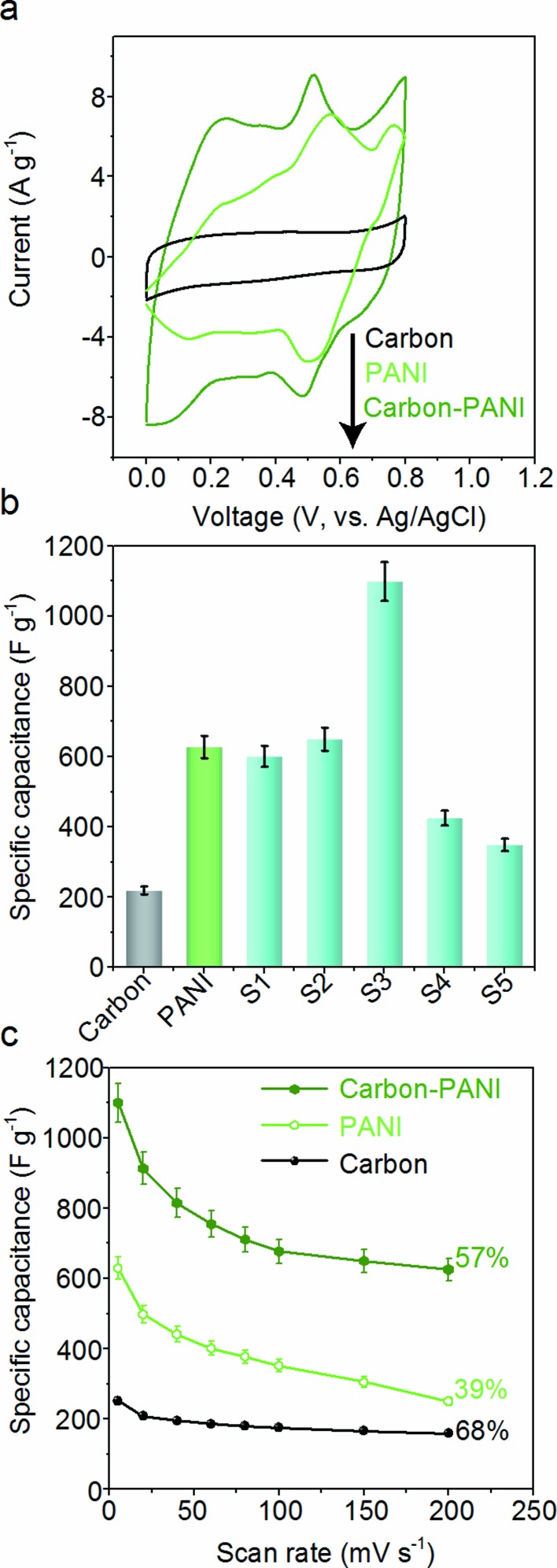
Supercapacitor characterization. (a) Comparative CVs of carbon, PANI and carbon–PANI (composition S3) samples at a scan rate of 5 mV s^–1^. (b) Comparison of the specific capacitance performances of carbon, PANI and carbon–PANI nanocomposites (S1–S5) at various time intervals. (c) Variation of capacitance with scan rate for carbon, PANI and carbon–PANI nanocomposite (composition S3) samples.

The capacitive behaviours of the carbon–PANI composites as electrode materials were investigated using cyclic voltammetry (CV) in a three-electrode configuration. Fig. 4a presents comparative CV curves of carbon, PANI and carbon–PANI composite (sample S3) samples in 1 M H_2_SO_4_ electrolyte in the potential window of 0.0 V to 0.8 V at a scan rate of 5 mV s^–1^. The comparative CV curves and capacitance variation with different scan rates for carbon, PANI and carbon–PANI nanocomposites (S1–S5) at scan rates from 5–200 mV s^–1^ are shown in Fig. S5.† The difference in the capacitance values between the carbon and the carbon–PANI composites is very remarkable. This fact can be easily observed from the CV curve shapes in Fig. 4a. Regardless of the PANI deposition time, the cyclic voltammetry (CV) curves of each of the carbon–PANI composites present a slightly sloped rectangular shape (Fig. 3a and S5†). The quasi-rectangular CV shape has two strong peaks, similar to a pseudocapacitor. These CV curves are attributed to the redox transitions of PANI (*i.e.*, the leucoemeraldine–emeraldine transition and emeraldine–pernigraniline transition).^47^ The maximum specific capacitance values obtained for these materials are 220, 629, 602, 651, 1100, 427 and 350 F g^–1^ for the carbon, PANI, S1, S2, S3, S4 and S5 samples, respectively (Fig. 4b). From this, it is clear that the capacitance value of the S3 sample is much higher than those of the other samples. This can be explained by the synergic cooperation between the redox active PANI shell and the EDLC supercapacitor (*i.e.*, the carbon core). Compared to previous reports, our samples show a very promising capacitance performance. A direct comparison of our results with the other reports in the literature is shown in Table S2.†

The variation of specific capacitance with scan rate is shown in Fig. 4c and S5.† Over the scan rate variation from 5 to 200 mV s^–1^, the capacitance values are found to decrease to 68%, 39%, 45%, 65%, 57%, 38% and 20% for carbon, PANI, S1, S2, S3, S4 and S5, respectively. Carbon–PANI composites with smaller shell thicknesses (samples S1–S3) show relatively good capacitance retention in comparison with bare PANI and the other carbon–PANI composites (S4 and S5). The optimized PANI shell coating provides high synergistic cooperation with the carbon core, improving the mechanical stability.

**Fig. 5:**
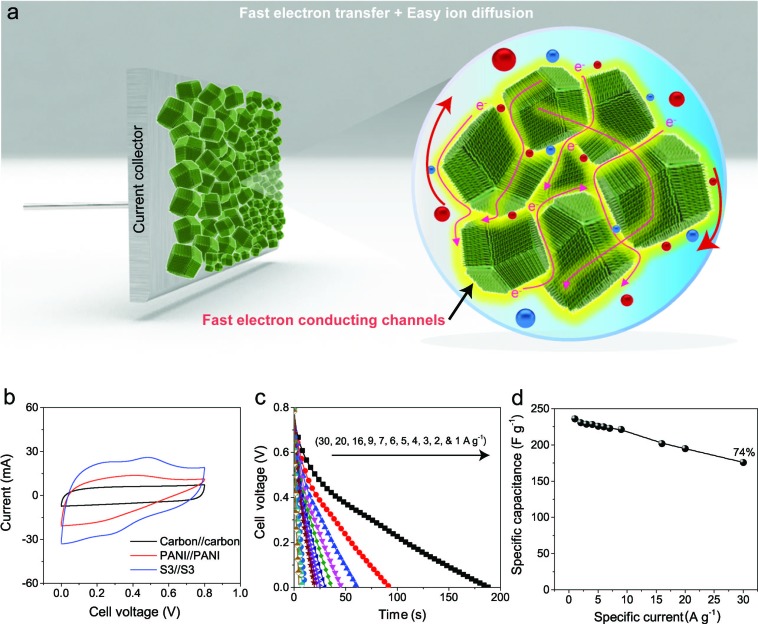
Electrochemical performances. (a) Unique multifaceted nanoarchitecture avoids the common problem of stacking that occurs with *e.g.* 1D CNTs or 2-D graphene, allowing for the easy diffusion of ions deep inside the material. Furthermore, the PANI nanorod arrays provide the ions with easy access to the carbon core, which leads to the enhanced synergy of these nanocomposites. The PANI nanorods also provide fast conducting channels (electron highways) for electrons to reach the collector surface. (b) Comparative CVs of carbon–PANI//carbon–PANI (S3), PANI//PANI, and carbon//carbon capacitors in 1 M H_2_SO_4_ electrolyte. (c) Discharge times for the carbon–PANI//carbon–PANI capacitor at various applied specific currents. (d) Plot of the variation of the specific capacitance with specific current.

To evaluate the true performances of the electrode materials, a two-electrode system is better than the three-electrode measurement. A comparative symmetric supercapacitor (SSC) study was carried out for carbon, PANI and carbon–PANI composite (sample S3) samples, as shown in Fig. 5, S6 and S7.† The comparative CVs of these three samples are shown in Fig. 5b. The charge–discharge (CD) studies were carried out at various applied specific currents (1, 2, 3, 4, 5, 6, 7, 9, 16, 20 and 30 A g^–1^). As seen in Fig. 5b, the discharge curves are very linear, and no *IR* drop is observed up to 30 A g^–1^. When the discharge profile curves for bare PANI (Fig. S7b†) and the carbon–PANI composite samples are compared with one another, it can be observed that bare PANI has a strong *IR* drop, which is unusual for such a highly conducting polymer. In the case of the PANI redox supercapacitor, the capacitance and charging capability strongly depend on the charging potential windows used.^27^ In the present study, in order to compare the cell performance for carbon, PANI and carbon–PANI composites, the potential window is fixed from 0.0 V to 0.8 V. However, bare PANI undergoes strong mechanical stress in the potential range of 0.6 to 0.8 V, which results in the observation of a big *IR* drop. Thus, polymer degradation and mechanical stress are critical factors responsible for the *IR* drop observed for the PANI sample. However, in the case of the carbon–PANI composite samples, the discharge profiles show no *IR* drop, which again confirms the advantage of the nanoporous carbon support. The specific capacitance values obtained are 236, 231, 229, 228, 226, 225, 223, 221, 202, 195 and 176 F g^–1^ at the applied specific currents of 1, 2, 3, 4, 5, 6, 7, 9, 16, 20 and 30 A g^–1^, respectively (Fig. 5c, the magnified view of the discharge profiles at high current densities is shown in Fig. S8†). A direct comparison of our results with previous reports is shown in Table S3.†

The observed decrease in capacitance with increasing applied specific current is a common issue in capacitor applications. Although some studies have reported promising performances for composites, retention at high scan rates is the main issue. For example, flexible graphene-PANI composites showed a retention of 82% when the applied current was varied from 0.3–6 A g^–1^.^34^ In another example, the capacitance of graphene-PANI composites decreased drastically, giving a retention of only 40% when the applied current was varied from 0.2–2 A g^–1^.^36^ This was probably due to the aggregation problem. During fast charge–discharge, electrolyte ions cannot intercalate/deintercalate across the deep void space. In the case of our composite samples, however, the comparative capacitance loss is only 6% when the applied specific currents are varied from 1 to 10 A g^–1^, as shown in Fig. 5d. Moreover, even when the applied specific current is greatly increased up to the very high value of 30 A g^–1^, the decrease in the capacitance is found to be only 26%. This indicates that our SSC with carbon–PANI is suitable for high retention in high power applications. As mentioned above, this unique core–shell composite shape can effectively avoid serious stacking of particles, as seen in Fig. 5a. The PANI network provides better conduction paths for easy transfer of the electrons. Also, the small-sized PANI nanorod array architecture provides good capacitance through redox reactions as well as easy access for the electrolyte ions to diffuse to the carbon core, which results in enhanced material utilization. On the other hand, the carbon support reduces the mechanical stress of PANI and enhances the stability of the polymer. In other words, each material effectively overcomes the other’s disadvantage, achieving a high and stable performance.

**Fig. 6:**
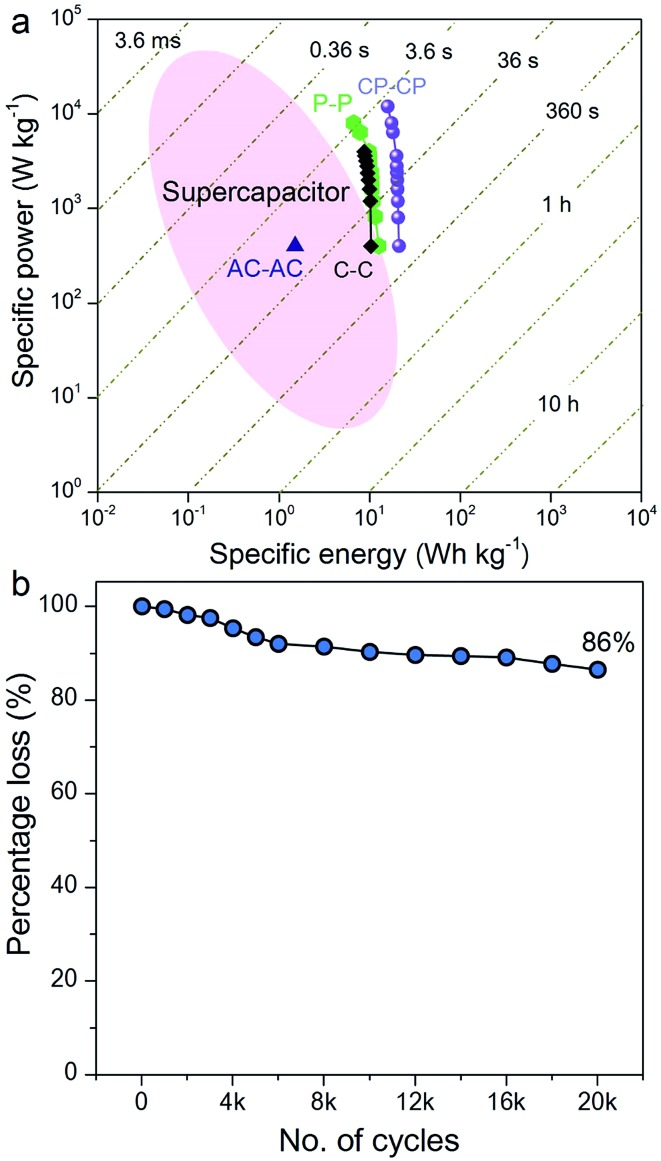
Electrochemical performances. (a) Ragone plot for symmetric supercapacitors based on activated carbon (AC–AC), carbon (C–C), PANI (P–P) and the carbon–PANI (CP–CP) nanocomposite. (b) Long-term cycling performance for the carbon–PANI core–shell nanocomposite.

The Ragone plot, which is a performance indicator for energy storage devices, was studied for our SSCs. Fig. 6a shows plots of specific energy *versus* specific power for three SSCs, using carbon, PANI and a carbon–PANI composite (sample S3). The specific power was found to increase with decreasing specific energy. The specific energy and the specific power obtained for the carbon sample are 10.26 W h kg^–1^ and 400 W kg^–1^, respectively. On the other hand, the PANI sample possesses a specific energy of 12 W h kg^–1^ and a specific power of 400 W kg^–1^ at the specific current value of 1 A g^–1^. For the carbon–PANI composite sample, the specific power and specific energy obtained at 1 A g^–1^ are 400 W kg^–1^ and 21 W h kg^–1^, respectively. Even at the very high specific current of 30 A g^–1^, high specific power and high specific energy are retained without much loss. More interestingly, when we compare our carbon sample for specific energy performance with a commercially available activated carbon material with a high surface area, the activated carbon sample has a remarkably low energy density (1.45 W h kg^–1^) in comparison with our carbon sample. This is owing to the activated carbon containing a large number of micropores compared to mesopores. Unlike in non-aqueous electrolyte,^48^ the micropores are inaccessible in aqueous electrolytes containing H_2_SO_4_ ions (due to heavy and large ions — H^+^ ions: 0.024 nm and SO_4_^–^: 0.24 nm), which results in decreased specific energies for these materials.^12^

The specific energy value obtained in this study surpasses previously reported SSCs using carbon fibre (CF)–PANI–RuO_2_ (10 W h kg^–1^),^47^ mesoporous carbon (9.6 W h kg^–1^),^49^ and other carbon materials (∼10 W h kg^–1^).^50^ Even when compared with other SSCs based on metal oxides, such as RuO_2_ (18.77 W h kg^–1^),^51^ Co(OH)_2_ (3.96 W h kg^–1^)^52^ and MnMoO_4_ (11 W h kg^–1^),^53^ our value is much higher than in these reports. It is even more interesting that our SSC shows a higher specific energy than some recent asymmetric supercapacitors (ASCs), such as NiMoO_4_//reduced graphene oxide (r-GO) (12.31 W h kg^–1^),^54^ Co(OH)_2_//graphene (11.9 W h kg^–1^)^55^ and Ni–Co oxy-hydroxide (17 W h kg^–1^).^56^

The electrochemical stability of the carbon–PANI composite was examined in 1 M H_2_SO_4_ aqueous electrolyte by galvanostatic CD measurements (Fig. 6b) at a current density of 5 A g^–1^. The electrochemical retention after 20 000 cycles showed a loss of only 14%, which is much smaller than in previous reports.^57^–^59^ The high stability of carbon–PANI originates from the synergistic cooperation of the carbon core and the PANI shell. This suggests that the carbon materials covered with optimized PANI nanorod arrays can sustain very long cycles of operation. This extraordinary stability combined with high specific power and high specific energy make this device a suitable candidate for future supercapacitor applications.

## Conclusion

We have demonstrated a unique synthetic approach for enhanced electrochemical performance by taking full advantage of the core–shell architecture. A uniform coating of PANI nanorods on a nanoporous carbon surface can be achieved by a simple oxidative polymerization. The multifaceted core–shell particles avoid the serious decrease in electrochemically active surface area that is caused by aggregation/stacking of active materials. The PANI nanorod arrays on the polyhedral carbon surface provide the electrolyte ions with high accessibility to the nanoporous carbon core. The SSC based on carbon–PANI electrodes achieves excellent capacitance performance compared with previous reports. The superior rate performance, even at high specific currents, is attributable to the deep void spaces being highly accessible to the ions. The high capacitance performances and good stabilities of our composites highlight the controlled synergy of the core–shell composites. This research provides a new pathway for the synthesis of 3-D core–shell architectures with different conductive polymers. By further optimizing the conductive polymers, higher performances and improved stabilities can be realized in the future.

## Supplementary Material

Supplementary information
