# Supplementary material for: Ultrahigh performance supercapacitors utilizing core–shell nanoarchitectures from a metal–organic framework-derived nanoporous carbon and a conducting polymer
Source: Chem Sci. 2016 Jun 10;7(9):5704–13. doi: 10.1039/c6sc01429a (PMC6022217; doi:10.1039/c6sc01429a)
Supplement: Supplementary file 1 [file SC-007-C6SC01429A-s001.pdf]

This journal is © The Royal Society of Chemistry 2016

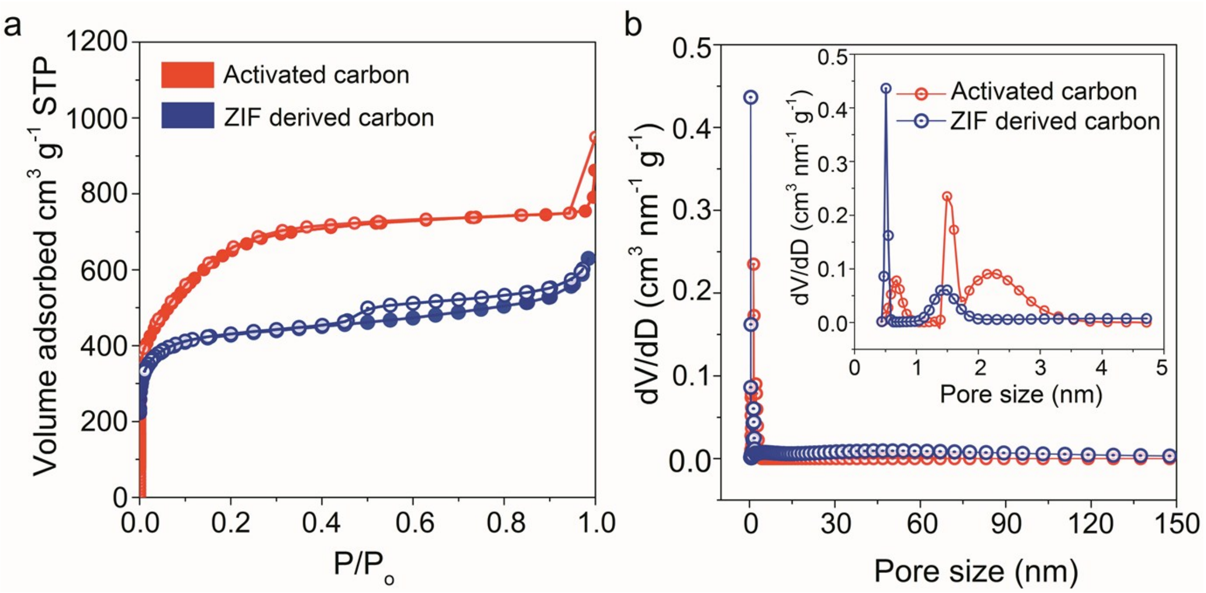

Figure S1 Comparative studies of (a) nitrogen adsorption-desorption isotherms, (b) NLDFT pore size distributions of commercially available activated carbon and our ZIF-derived carbon samples.

Table S1. Summary of surface areas for activated carbon and ZIF-derived nanoporous carbon.

Activated carbon	ZIF-derived nanoporous carbon
Surface area: 2,370 $m^2 \cdot g^{-1}$<br>Micropore surface area: 1,743 $m^2 \cdot g^{-1}$<br>Relative micropore ratio: 73.5%	Surface area: 1,610 $m^2 \cdot g^{-1}$<br>Micropore surface area: 960 $m^2 \cdot g^{-1}$<br>Relative micropore ratio: 59.6%

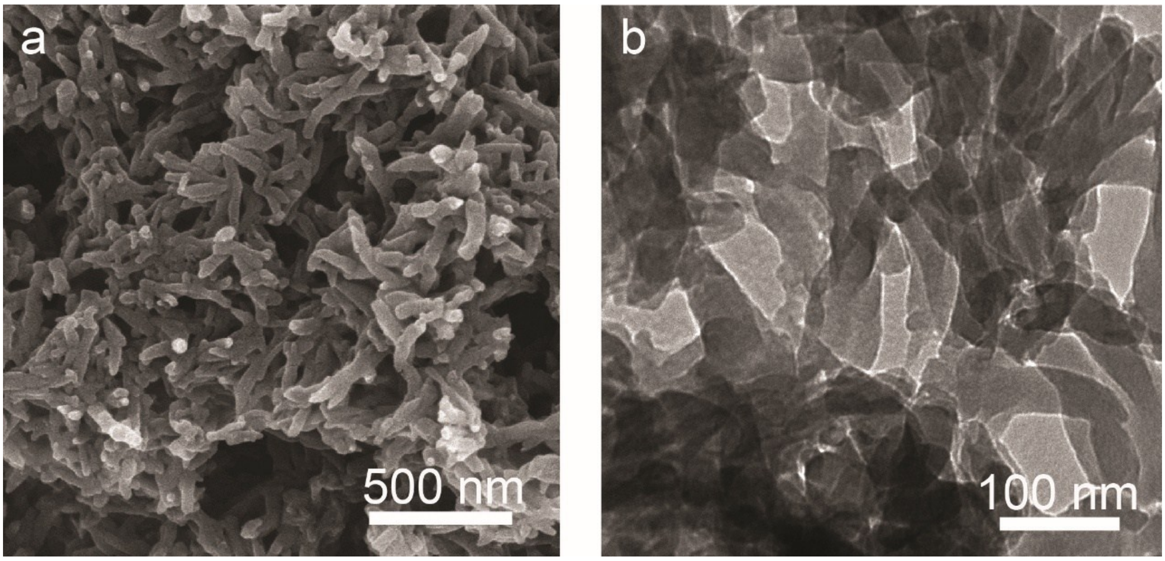

Figure S2 (a) SEM and (b) TEM images of bare PANI sample after 6 h reaction time.

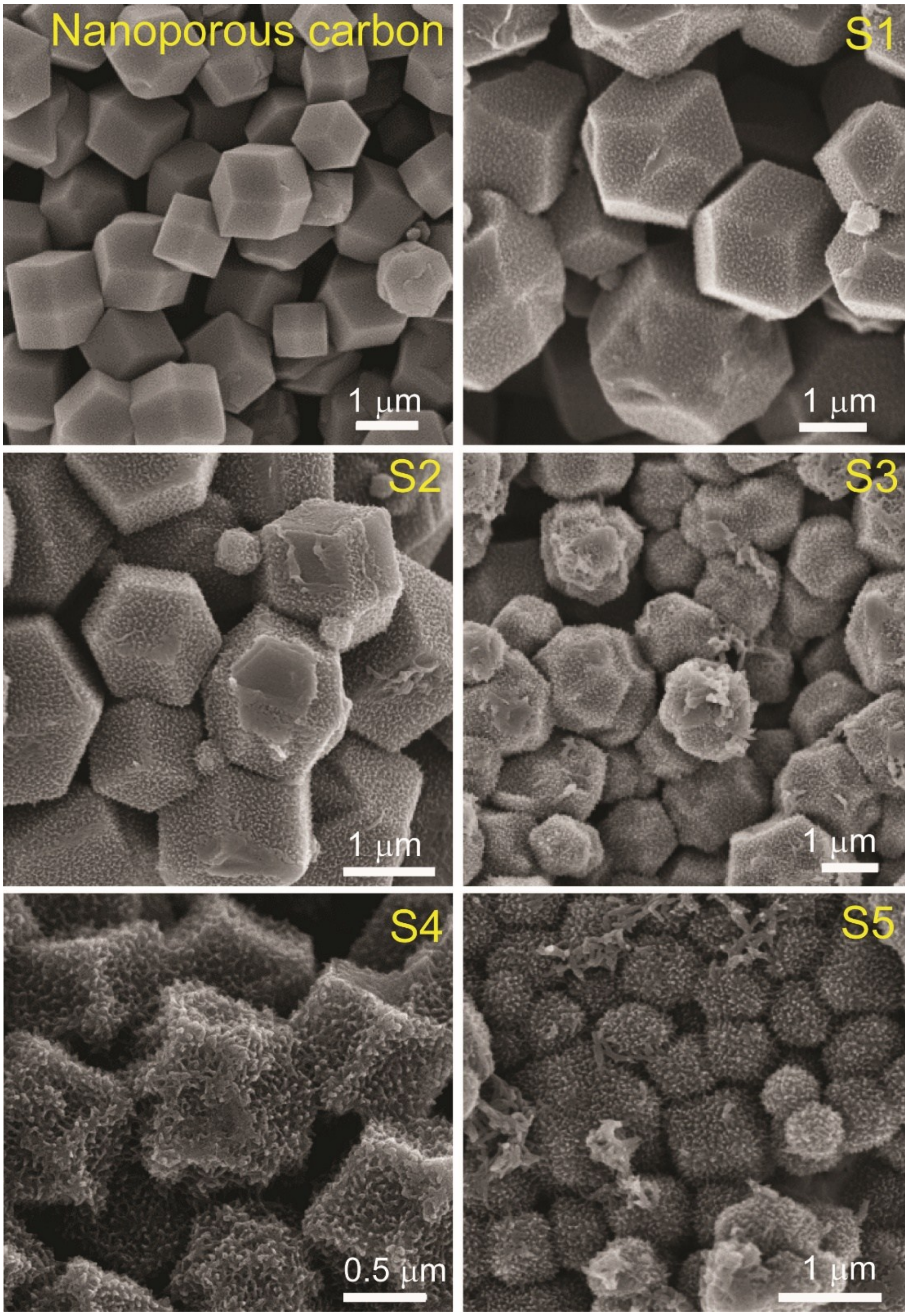

Figure S3 The low magnified SEM images of nanoporous carbon and carbon-PANI composites (S1, S2, S3, S4 and S5), showing uniform coating of PANI and dispersion on the nanoporous carbon surface.

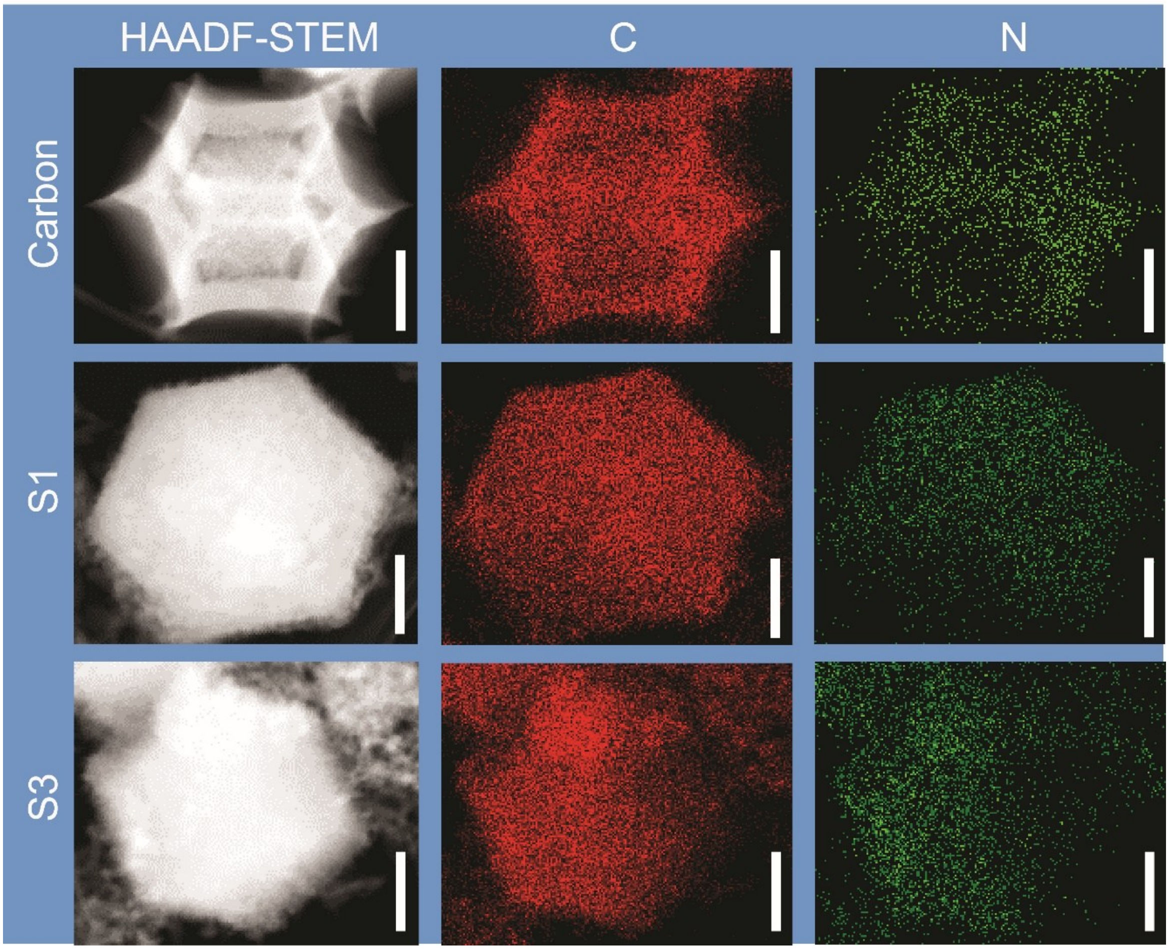

Figure S4 High-angle annular dark field-scanning TEM (HAADF-STEM) images and elemental mapping of carbon and nitrogen for carbon sample and different carbon-PANI composites (samples S1, S3). The scale bars are 300 nm.

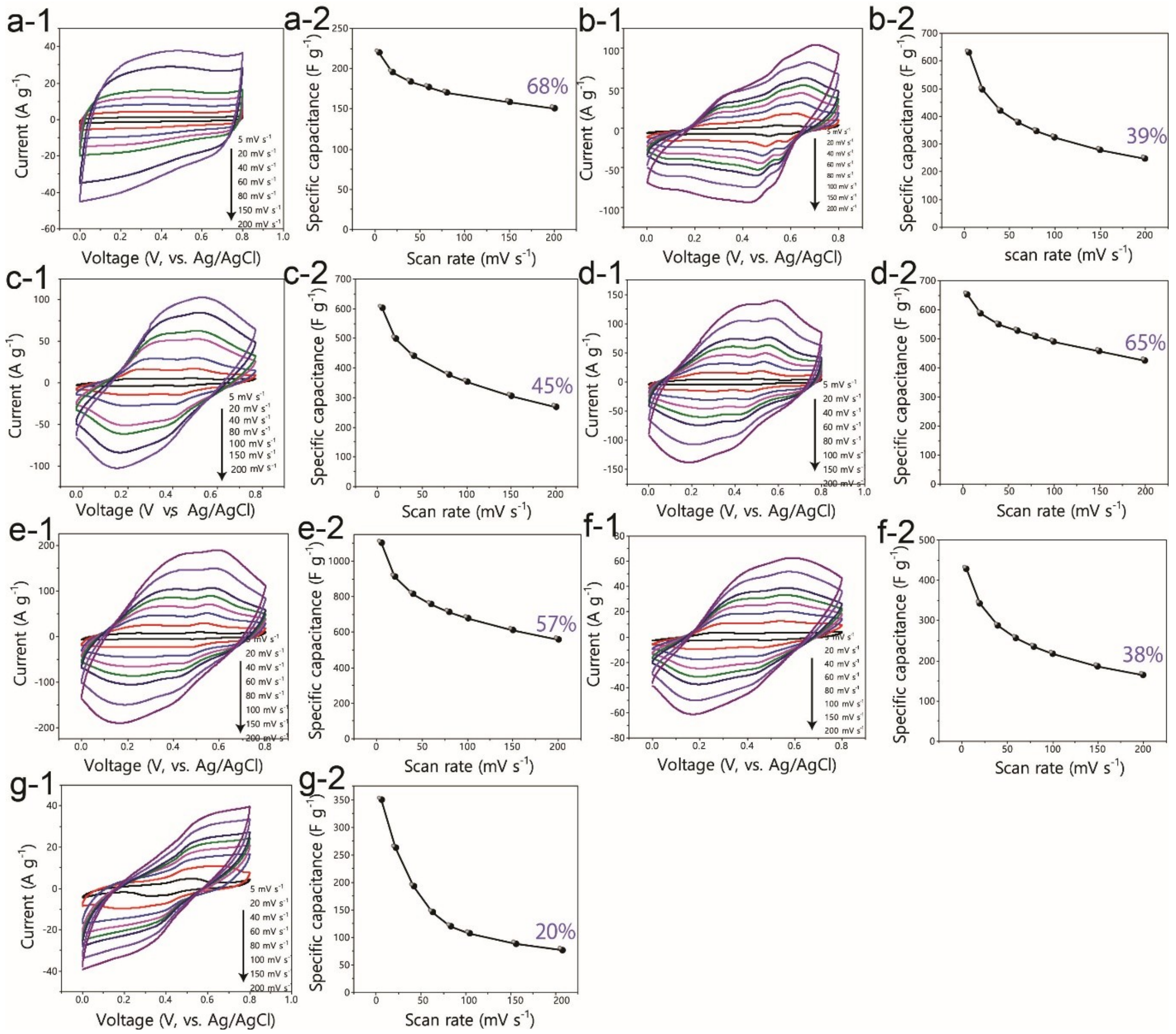

Figure S5 CV curves and variation of specific capacitance with different scan rates for each sample using the three-electrode system: (a) carbon, (b) PANI, (c) S1, (d) S2, (e) S3, (f) S4, and (g) S5.

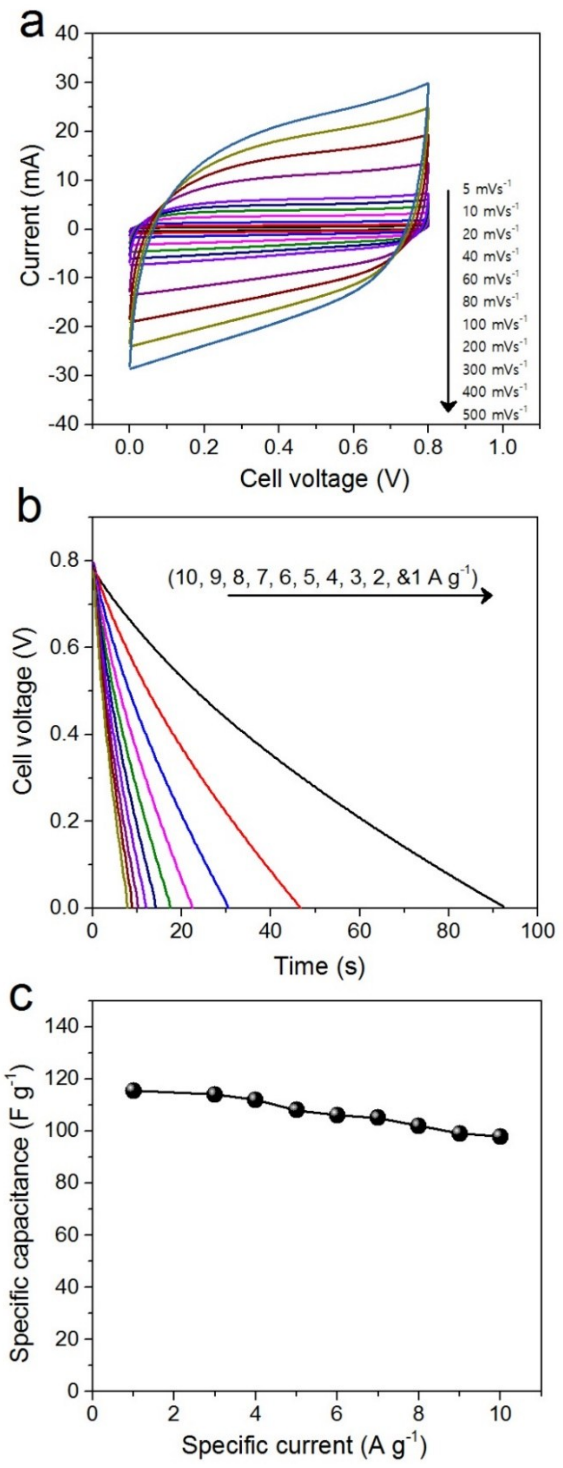

Figure S6 Two-electrode data for carbon sample. (a) CVs at various scan rates, (b) charge-discharge (CD) study at various applied current densities, and (c) variation of specific capacitance with specific current.

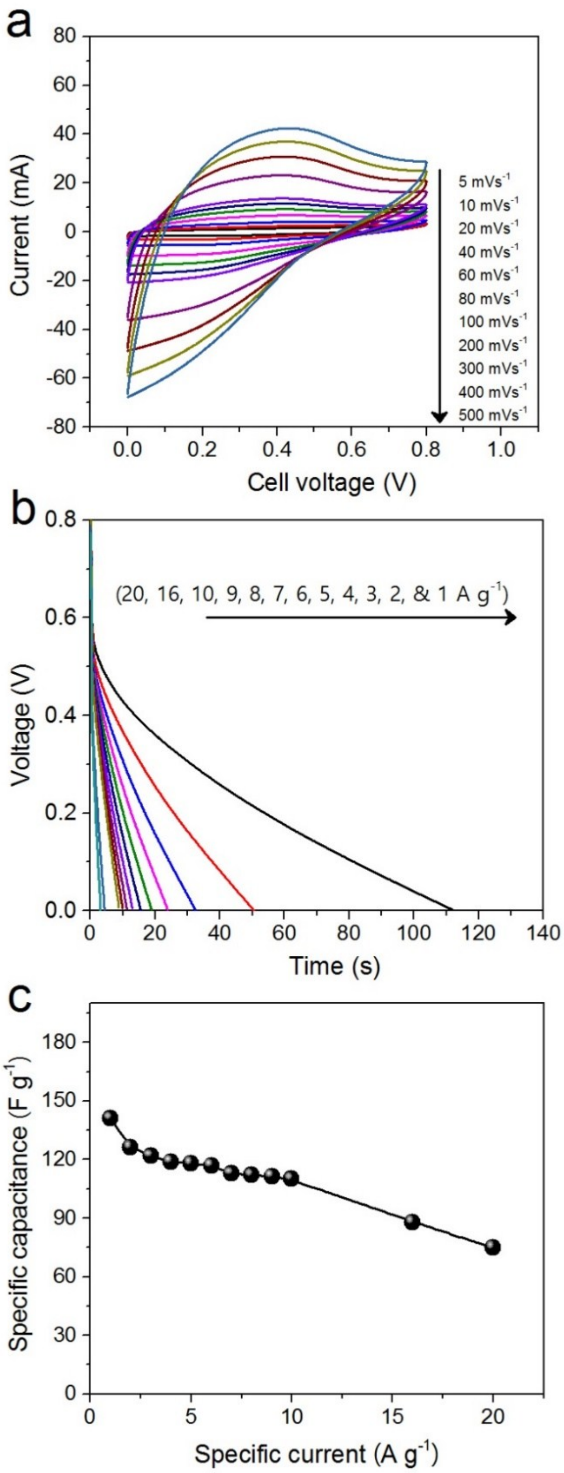

Figure S7 Two-electrode data for PANI sample. (a) CVs at various scan rates, (b) CD study at various applied current densities, and (c) variation of specific capacitance with specific current.

Note for Figures S6 and S7

In order to evaluate the SSC performance of carbon//carbon and PANI//PANI, cyclic voltammetry (CV) and charge-discharge (CD) studies were carried out. **Figure S6a** shows CVs for carbon//carbon SSC at various scan rates from 5-500 $mV\cdot s^{-1}$. The CVs are mirror symmetric and rectangular in shape. The CV shape is retained very well even at the higher scan rates. The CD studies were carried out at various applied specific currents from 1-10 $A\cdot g^{-1}$. The respective discharge curves are as shown in **Figure S6b**. The discharge capacitance shows good retention of 85%, even when the applied specific current values increased to 10 $A\cdot g^{-1}$ (**Figure S6c**). Thus, the nanoporous carbon sample shows good retention capability at the high scan rates. In the case of the PANI sample, the CV shape is quasi-rectangular at lower scan rates (5-100 $mV\cdot s^{-1}$). With increasing scan rate (100-500 $mV\cdot s^{-1}$), however, the shapes are distorted (**Figure S7a**). The distorted shape represents instability of the sample at the higher scan rates. Furthermore, the CD studies also show an *IR* drop from 0.8 to 0.6 V (**Figure S7b**). The variation of capacitance with specific current is shown in **Figure S7c**. The capacitance retention is found to be 53 %, which is much lower compared to the carbon-based SSC.

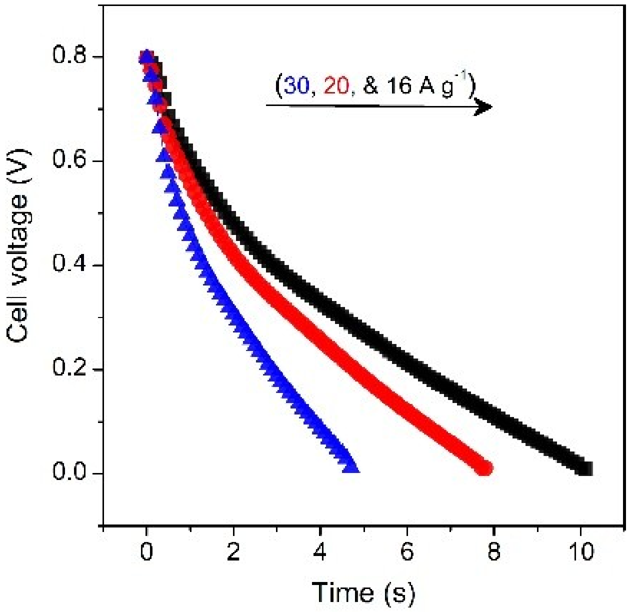

Figure S8. Variation of discharge times with relatively higher current densities at 30, 20, and 16 A.g^{-1}.

Table S2. Comparison of our specific capacitance value with various literature reports for carbon-PANI composites using the three-electrode system.

Method	Electrode	Electrolyte	Specific capacitance ($F \cdot g^{-1}$)	Scan rate ($mV \cdot s^{-1}$)	Specific current ($A \cdot g^{-1}$)	Voltage window (V)	Weight loading ($mg \cdot cm^{-2}$)	Ref.
**Carbon nanotube-PANI/carbon fiber-PANI composites**								
Electrochemical polymerization	SWCNT-PANI	H_2SO_4 (1 M)	485	-	5 $mA \cdot cm^{-2}$	0.0-0.7	-	[S1]
Vapor deposition polymerization	CNF-PANI	H_2SO_4 (1 M)	264	5	-	0.0-0.8	-	[S2]
In-situ polymerization	MWCNT-PANI	H_2SO_4 (0.1 M)	560	1	-	0.0-1.0	-	[S3]
Oxidative polymerization	CNT-PANI	KOH (6 M)	780	1	-	(-0.7)-0.3	4	[S4]
Oxidative polymerization	CNF-PANI	H_2SO_4 (1 M)	638	-	2	0.0-0.8	0.35	[S5]
**Graphene-PANI/graphene oxide-PANI/reduced graphene oxide-PANI composites**								
Oxidative polymerization	rGO-PANI	H_2SO_4 (1 M)	286	5	-	(-0.1)-0.9	0.5	[S6]
Oxidative polymerization	Graphene-PANI	H_2SO_4(1 M)	250	10		(-0.2)-1.0	-	[S7]
Oxidative polymerization	Graphene-PANI	H_2SO_4 (1 M)	257	-	0.1	(-0.2)-0.8	0.01	[S8]
Electropolymerization	Graphene-PANI	H_2SO_4 (1 M)	233	2	-	(-0.2)-0.8	0.58 g cm^{-3} (volume density)	[S9]
In-situ polymerization	Graphene-PANI	H_2SO_4 (1 M)	1126	1	-	(-0.2)-0.6	-	[S10]
In-situ polymerization	GNS-PANI NF	KOH (6 M)	1046	1	-	(-0.7)-0.3	5	[S11]
Rapid mixture polymerization	Graphene-PANI	H_2SO_4 (1 M)	475	-	0.4	0.0-0.8	0.75	[S12]
In-situ polymerization	Graphene-PANI	H_2SO_4 (2 M)	480	-	0.1	(-0.2)-0.8	-	[S13]
**Porous carbon-PANI composites**								
Electrochemical polymerization	Porous carbon-PANI	H_2SO_4 (1 M)	180	-	1 mA	0.0-0.6	2.9	[S14]
In-situ polymerization	OMC-PANI	KOH (30 wt.%)	747	-	0.1	0.0-0.9	1	[S15]
Oxidative polymerization	MC-PANI SS	H_2SO_4 (1 M)	940	-	0.5	(-0.2)-0.7	2.5	[S16]
Electrochemical polymerization	Macroporous carbon-PANI	H_2SO_4 (1M)	1198	-	1	(-0.2)-0.8	-	[S17]
Oxidative polymerization	Nanoporous carbon PANI	H_2SO_4 (1M)	1100	5	-	0.0-0.8	0.5	Present work

PANI- Polyaniline; SWCNT- single walled carbon nanotube; MWCNT- multi walled carbon nanotube; rGO- reduced graphene oxide; GNS- Graphene nanosheets; OMC- ordered mesoporous carbon; NF-nickel foam; SS- stainless-steel grid.

Table S3. Comparison of specific capacitance values with literature reports for various carbon-PANI composites using the two-electrode system.

Method	Electrode	Electrolyte	Specific capacitance ($F \cdot g^{-1}$)	Scan rate ($mV \cdot s^{-1}$)	Specific current ($A \cdot g^{-1}$)	Voltage window (V)	Weight loading ($mg \cdot cm^{-2}$)	Ref.
Dilute polymerization	r-GO-PANI	H_2SO_4 (1 M)	385	-	0.5	0.0-1.0	0.25	[S18]
Interfacial polymerization	Graphene-PANI	H_2SO_4 (1 M)	210	-	0.3	0.0-0.8	0.76 $g \cdot cm^{-3}$ (Volume density)	[S19]
Dilute polymerization	Graphene oxide-PANI	H_2SO_4 (1 M)	555	-	0.2	0.0-0.8	-	[S20]
Dilute polymerization-atomic layer deposition	CC/PANI-RuO_2	H_2SO_4 (1 M)	710	5	-	0.0-0.7	~1	[S21]
Oxidative polymerization	Nanoporous carbon-PANI	H_2SO_4 (1 M)	236	-	1.0	0.0-0.8	0.5	Present work

CC- carbon cloth; RuO_2- ruthenium oxide

Supporting References

[S1] V. Gupta, and N. Miura, *Electrochim. Acta*, 2006, **52**, 1721-26.

[S2] J. Jang, J. Bae, M. Choi, and S. H. Yoon, *Carbon*, 2005, **43**, 2730-36.

[S3] Y. Zhou, Z. Y. Qin, L. Li, Y. Zhang, Y. L. Wei, L. F. Wang and M. F. Zhu, *Electrochim. Acta*, 2010, **55**, 3904-08.

[S4] J. Yan, T. Wei, Z. Fan, W. Qian, M. Zhang, X. Shen and F. Wei, *J. Power Sources*, 2010, **195**, 3041-45.

[S5] X. Yan, Z. Tai, J. Chen, and Q. Xue, *Nanoscale*, 2011, **3**, 212-16.

[S6] R. R. Salunkhe, S. H. Hsu, K. C. W. Wu and Y. Yamauchi, *ChemSusChem*, 2014, **7**, 1551-56.

[S7] N. A. Kumar, H. J. Choi, Y. R. Shin, D. W. Chang, L. Dai and J. B. Baek, *ACS Nano*, 2012, **6**, 1715-23.

[S8] Z. F. Li, H. Zhang, Q. Liu, L. Sun, L. Stancia and J. Xie, *ACS Appl. Mater. Interfaces*, 2013, **5**, 2685-91.

[S9] D. W. Wang, F. Li, J. Zhao, W. Ren, Z. G. Chen, J. Tan, Z. S. Wu, I. Gentle, G. Q. Lu and H. M. Cheng, *ACS Nano*, 2009, **3**, 1745-52.

[S10] H. Wang, Q. Hao, X. Yang, L. Lu, and X. Wang, *Nanoscale*, 2010, **2**, 2164-70.

[S11] J. Yan, T. Wei, B. Shao, Z. Fan, W. Qian, M. Zhang and F. Wei, *Carbon*, 2010, **48**, 487-93.

[S12] X. Yan, J. Chen, J. Yang, Q. Xue and P. Miele, *ACS Appl. Mater. Interfaces*, 2010*,* **2**, 2521-29.

[S13] K. Zhang, L. L. Zhang, X. S. Zhao and J. Wu, *Chem. Mater.*, 2010, **22**, 1392-1401.

[S14] W. C. Chen, T. C. Wen and H. Teng, *Electrochim. Acta*, 2003, **48**, 641-49.

[S15] L. Li, H. Song, Q. Zhang, J. Yao and X. Chen, *J. Power Sources*, 2009, **187**, 268-74.

[S16] Y. G. Wang, H. Q. Li, Y. Y. Xia, *Adv. Mater.*, 2006, **18**, 2619-23.

[S17] L. L. Zhang, L. Shi, J. Zhang, P. Guo, J. Zheng and X. S. Zhao, *Chem. Mater.*, 2010, **22**, 1195-1202.

[S18] Y. Meng, K. Wang, Y. Zhang and Z. Wei, *Adv. Mater.*, 2013, **25**, 6985-90.

[S19] Q. Wu, Y. Xu, Z. Yao, A. Liu and G. Shi, *ACS Nano*, 2010, **4**, 1963-70.

[S20] J. Xu, K. Wang, S. Z. Zu, B. H. Han and Z. Wei, *ACS Nano*, 2010, **4**, 5019-26.

[S21] C. Xia, W. Chen, X. Wang, M. N. Hedhili, N. Wei and H. N. Alshareef, *Adv. Energy Mater.*, 2015, **5**, 1401805.
